# EEG for Diagnosis of Adult ADHD: A Systematic Review With Narrative Analysis

**DOI:** 10.3389/fpsyt.2020.00871

**Published:** 2020-08-25

**Authors:** Marios Adamou, Tim Fullen, Sarah L. Jones

**Affiliations:** ^1^School of Human & Health Sciences, University of Hudderfield, West Yorkshire, United Kingdom; ^2^Adult ADHD & Autism Service, South West Yorkshire Partnership NHS Foundation Trust, Wakefield, United Kingdom

**Keywords:** electroencephalogram, attention deficit hyperactivity disorder, diagnosis, neuroimaging, clinical psychology

## Abstract

**Background:**

Attention deficit hyperactivity disorder is a common neurodevelopmental disorder characterized by symptoms of inattention, hyperactivity and or impulsivity. Since the development of the concept, a reliable biomarker to aid diagnosis has been sought. One potential method is the use of electroencephalogram to measure neuronal activity. The aim of this review is to provide an up to date synthesis of the literature surrounding the potential use of electroencephalogram for diagnosis of attention deficit hyperactivity disorder in adulthood.

**Methods:**

A search of PsycINFO, PubMed, and EMBASE was undertaken in February 2019 for peer-reviewed articles exploring electroencephalogram patterns in adults (18 years with no upper limit) diagnosed with attention deficit hyperactivity disorder.

**Results:**

Differences in electroencephalogram activity are potentially unique to adult attention deficit hyperactivity disorder populations. Strongest support was derived for elevated levels of both absolute and relative theta power, alongside the observation that alpha activity is able to typically differentiate between adult attention deficit hyperactivity disorder and normative populations.

**Conclusions:**

Electroencephalogram can have a use in clinical settings to aid adult attention deficit hyperactivity disorder diagnosis, but areas of inconsistency are apparent.

## Background

Having discovered that the mode of function of the human central nervous system is based on electric activity (1, 2) the invention of electroencephalography (EEG) and its first applications in humans (3) provided the possibility of analyzing the brain at its core functional level. Establishing a method that monitors the electrical activity of the cortex was revolutionary in elucidating functional aspects of common neurological conditions (4). Since then, EEG has been most commonly used in the diagnosis and management of epilepsy due to its capacity to discern both the classification and localization of seizures (5). EEG has also proven valuable in diagnosing and monitoring other forms of neuropathology including traumatic brain injury, dementia, encephalitis, brain tumors, and sleep disorders (6).

Alongside its use to better understand organic pathology, developments in its application saw an emphasis upon mapping neural correlates believed to be associated with psychiatric disorders. For instance, in schizophrenia, EEG patterns were studied as early as the 1960s (7), with evidence demonstrating reduced alpha activity with greater beta and slow wave delta voltage in quantitative EEG (qEEG) (8). Further, Knott & Lapierre (1987, 9) found deactivation in the right hemisphere in patients with major depression versus controls using EEG, and global waveform patterns between patients with post-traumatic stress disorder and normal controls have been investigated, finding resting state EEG demonstrated reduced complexity of global waveforms in the experimental group (10). For bipolar disorder, EEG analysis has been able to classify type I and type II bipolar disorder in adolescents (11). In characterizing social anxiety disorder, Harrewijn, Van der Molen, & Westenberg (12) found a negative delta-beta correlation in highly anxious participants comparative to those with lesser anxiety. In a similar review as this one, EEG abnormalities were found to be more severe in early-onset Alzheimer Disease patients (13). However, despite the potential usefulness of these findings, EEG has been notably absent from application to clinical practice.

### EEG and ADHD

More recently, EEG has been used to identify possible biomarkers in neurodevelopmental disorders. For example, it has recently been suggested that EEG can be used for the diagnosis of Autism Spectrum Disorder (ASD) (14) and even measure its severity (15). In terms of ADHD, over the past decade researchers have used EEG to identify a number of localized changes in children with ADHD, purporting to differentiate cortical activity from ADHD and non-ADHD children (16). In these studies, the most commonly reported difference is in the Theta/Beta Ratio (TBR), the elevation of which is associated with childhood ADHD. Other differential biomarkers which are reported include increased alpha and resting state theta activity (17).

However, despite the proliferation of research into EEG and childhood ADHD, a significantly smaller number of studies have sought to determine neural correlates and biomarkers in adulthood. These are the ones we are focusing on in this review. The possibility of differentiating adult ADHD specific biomarkers remains a potentially important means of diagnosing the condition in this group and would be considered a welcome development by clinicians (18). Given that clinical presentation of ADHD appears to evolve and diminish across the developmental course, it is important to ascertain if any functional differences exist specific to the adult population. This is of pressing importance, considering that ADHD assessment is largely dependent on subjective patient report and clinical observations. Should EEG be able to be applied in clinical practice as a means to aid diagnosis, it would provide a potentially noninvasive and economical method with which to objectify the assessment process. Although there are a good number of studies for the use of EEG in adult ADHD, there is only one published review to date. Lenartowicz and Loo (2014, 19) explored the use of EEG in diagnosing adult ADHD, concluding that EEG was not an appropriate diagnostic tool but has a potentially promising future. This however requires revising due to passage of time and particularly due to the increase in numbers of studies in the last five years.

This review synthesized research which used EEG to map cortical activity in adults with a formal diagnosis of ADHD. The aims of this review was to (i) discern whether there are any differences between resting state and event related potential (ERP) cortical activity in the adult ADHD brain from the normative population, and (ii) to ascertain whether this evidence may lend itself to position EEG in clinical practice to aid diagnosis.

## Methods

### Inclusion Criteria

Quantitative reports of EEG used in the adult ADHD population were included. Criteria stipulated that studies appear in peer-reviewed journals in English language. Studies must present EEG neurophysiological outcome data, and concern subjects with a confirmed diagnosis of ADHD. Case studies are excluded. Due to the relative infancy of research in this area studies of controlled and noncontrolled design are included. No time limits were applied to the search parameters.

This review considers all outcomes relating to primary symptoms of ADHD. Studies may or may not include follow-up of various lengths. For the purposes of the review, adulthood is defined from 18 years old with no upper limit. Participants must have met diagnostic criteria for ADHD confirmed either by clinical assessment, or as reported using a cut off on at least one recognized diagnostic tool. Studies were excluded if ADHD was a secondary diagnosis, if the focus was on psychopharmacological or neuro-feedback outcomes. Participants could also report any comorbidity commonly associated with ADHD (e.g. bipolar); however, any diagnosis that might take precedent (e.g. moderate-severe intellectual disability) was excluded here. Studies were assessed independently by two authors; any uncertainty of appropriateness was discussed in order to achieve a consensus.

### Data Extraction

Where reported, the following data were extracted from each article: author, year of publication, country, methodological approach, demographics, number of subjects, and main findings.

### Study Quality Assessment

The study quality was assessed using a standardized quality-rating tool. The Downs and Black (20) method provides a framework for assessing the quality of randomized and nonrandomized trials. The quality rating checklist considers the following areas: (i) reporting (ii) external validity, (iii) bias, (iv) confounding variables, and (v) power. Four of the five subscales demonstrated high internal consistency and the quality index correlates highly with existing standardized appraisal tools for assessing randomized studies (*r* = .90).

Papers rated as poor quality were included in data extraction as our primary search objective was of including all available research. Poor quality papers were notably limited in the quality of reporting and internal validity due to lack of controlling for confounding variables. The tool was employed using a modification in order to be applicable to the nature of the studies included in the review. Therefore maximum score was 22, classified as excellent were scores of 18–22, good 15–17, fair 12–14, and poor <11. Quality was assessed by one author; any uncertainty of rating was discussed with other authors in order to achieve a consensus. While quality rating is useful in giving an overall evaluation of the literature, it was not a used as an exclusion criterion.

### Search Strategies

The following terms were used in the title, ADHD and EEG; ADD and EEG, ADD and Encephal*, ADHD and Encephal*, Encephal* and Hyperkin*, Hyperkin*, and EEG and adult* in the abstract field. No time limits were applied to the search.

**Figure 1:**
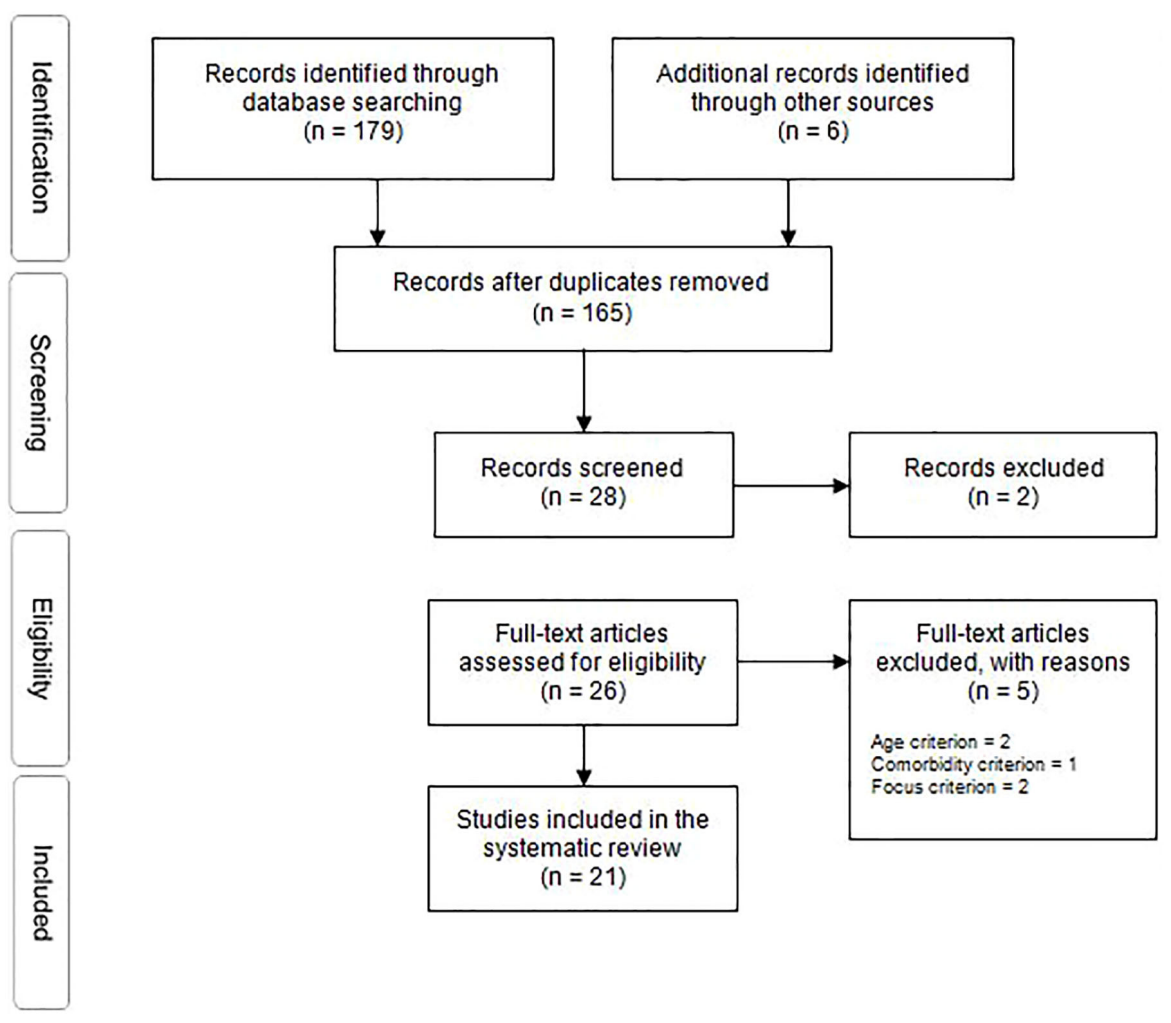
PRISMA flow diagram (21).

In order to identify relevant primary studies, databases germane to the research area (PsycINFO, PubMed, and EMBASE) were searched. Final searches were conducted in February 2019. In addition, hand searches of published systematic reviews, and of key journal publications were undertaken. Finally, following the application of the inclusion criteria, citation searches concluded the search. The initial search yielded 179. After duplicates were removed, studies were assessed for eligibility using PICOS methodology and exclusion-inclusion criteria was applied. Finally, 21 studies were included in this review (**Figure 1**; **Table 1**).

**Table 1:** Characteristics of the studies included in the review.

Study First author, year, country	Methodology	Demographics	Sample Size	Main findings	Quality rating
Biederman, 2017, US (22)	Collected EEG while the subjects were performing Go/NoGo task. Applied spatio-temporal BNA analysis	Nonmedicated right-handed 18 to 55 year old adults of both sexes with and without a DSM-IV diagnosis of ADHD	*n* = 63 ADHD = 34 (13 female), control = 29 (14 female)	BNA methodology demonstrated a high discriminative capacity between ADHD patients and controls based on functional brain connectivity	18 (excellent)
Bresnahan, 2002, Australia (23)	Resting state eyes open 2-min condition. 24-channel digital signal-processing at 17 sites. Between groups comparison	Excluded comorbid psychiatric disorder, neurological impairment, and substance disorder. Mean age 31.5 years	*n* = 150 ADHD = 50, typical development with elevated theta = 50, control = 50 (50% female each group). Matched on age	ADHD group differed from both the non-ADHD and the control groups on the basis of elevated theta activity. ADHD and control groups did not differ in beta activity, but relative theta was reduced and relative beta power was elevated in the non-ADHD group compared with both the ADHD and control groups	17 (good)
Broyd, 2011, UK (24)	66 channel electrode measuring at rest and task condition. Within and between group analysis	Excluded diagnosed neurological disorder, consumed caffeine within the 2 h prior to testing, had used any psychotropic substance (illicit or otherwise) in the 24 h prior to testing, and/or more than once a month in the previous six months. Mean age 22.25 years	*n* =50 severe ADHD = 25, mild ADHD = 25	Deactivation of VLF EEG power between the rest and task condition for the whole sample, deactivation sources were different for high and low ADHD groups: in the low ADHD group attention-induced VLF EEG deactivation was most significant in medial prefrontal regions while for the high ADHD group this deactivation was predominantly localized to the temporal lobes	13 (fair)
Gonen-Yaacovi, 2016, Israel (25)	Trial-by-trial EEG response variability to visual and auditory stimuli while subjects' attention was diverted to an unrelated task at the fixation cross	Mean age 25 years. ADHD individuals taking stimulants were instructed to abstain from medication for at least 24 h before participation. Confirmed diagnosis of ADHD. Three ADHD participants also showed comorbidity. Groups were matched on general intelligence	*n* = 34 ADHD = 17, control = 17	Larger response variability in the ADHD group for visual and auditory stimuli compared to controls	15 (good)
Hale, 2010, US (26)	40 electrodes using the International 10/20 locations system. EEG recording at 2 baselines lasting 5 min each eyes open and eyes closed and a cognitive activation condition. Within and between group analysis.	Excluded if taking psychoactive medication, reported neurological disorder, or diagnosis of schizophrenia or autism, or IQ < 80.	*n* =139 ADHD = 35, 104 = control	Adults with ADHD showed pronounced rightward beta asymmetry in inferior parietal regions (P8–P7) during CPT	17 (good)
Hale, 2014, US (27)	*	*	*	Abnormal rightward inferior parietal beta asymmetry in adults with ADHD during the CPT, Rightward beta and theta asymmetry across inferior, superior, and temporal-parietal brain regions, and showed that rightward parietal asymmetry in ADHD was atypically associated with multiple cognitive tests	17 (good)
Hasler, 2016, Switzerland (28)	Functional mechanisms of attention deficits explored using activities associated with bottom-up attentional cueing (temporal and spatial orienting of attention) and top-down control (conflict resolution) Comparison between eyes open and eyes closed conditions, and between groups of subjects. 19 silver-chloride electrodes were applied according to the International 10–20 system	ADHD group mean age 40.05 years. Excluded nonnormal visual acuity, history of major medical disorders, head injury, neurological disorders, and alcohol or drug abuse. Other current psychiatric disorders were exclusion criteria for ADHD patients	*n* =41 ADHD = 21, control = 20	ADHD reduced P3 amplitude, preparatory activation in both alpha and beta bands, as well as flattened target-related posterior alpha and beta responses	17 (good)
Herrmann, 2010, Germany (29)	Eriksen flanker task while recording the neural activity with 26 scalp EEG electrodes	Mean age of 33 years. Patients discontinued all medication 3 days prior. Age was included as a control variable. ADHD patients were excluded if they had a current Axis I mood, substance-related, psychotic, or anxiety disorder and/or a concurrent Axis II disorder according to DSM-IV. Or had a history of dependence on illegal drugs or alcohol, as well as any current psychotropic medication	*n* = 68 ADHD = 34, control = 34. For statistical analyses, authors separated both groups according to their age median into a younger and an elderly subgroup	Reduced Pe amplitudes, but also reduced ERN values, in ADHD patients. Importantly, these differences as well as the deficits in behavioral performance were mainly detectable in the younger subsample, but not in the elderly subsample	19 (excellent)
Kitsune, 2015, UK (30)	Resting-state EEG power or GFS between recordings made at the beginning and end of testing session. Within and between group comparison. 62 channel DC-coupled recording system. 1.5-h cognitive test battery. 2-min eyes open recording performed before and after	ADHD mean age, 18.70 years. Excluded if, IQ < 70, reported autism, epilepsy, learning difficulties, brain disorders and any genetic or medical disorder associated with externalizing behaviors that might mimic ADHD	*n* =161 ADHD = 76, control = 85	The ADHD group had higher delta and theta power at time-1, but not at time-2, whereas beta power was elevated only at time-2. Significant IQ effects	15 (good)
Koehler, 2009, Germany (31)	Two EEG readings over 5-min intervals during eyes-closed resting period. 21 electrodes placed according to the international 10–20 system. Within and between group comparisons	34 patients between 18 and 55 years of age, mean age 33.26 years. 1:1 ratio male-female	*n* = 68 ADHD = 34, control = 34	The ADHD patients showed a significant increase of absolute power density in alpha and theta bands. No differences were found for beta activity	18 (excellent)
Leroy, 2018, Belgium (32)	ERP study. Oddball paradigm using implicit navigational images and analyzed EEG dynamics with swLORETA inverse modeling of the evoked potential generators to study cortical processing. Unipolar EEG recordings were performed from 128 electrodes on a cap	Mean age was 38 years. Eight out of the 14 ADHD patients were medication-naïve and 6 patients were on medication (but not actually receiving treatment at the time of the recording). Exclusion criteria for both groups were a seizure disorder, head injury affecting the central nervous system, mental retardation, and sensory deficits. Comorbid disorders were not excluded; 4 out of the 14 ADHD participants had one comorbid disorder	*n* = 28 ADHD = 14, control = 14	Visual attention task produced decrease in neural networks. Differentiation of delta-theta in executive function, specifically in visual tasks. Overall, findings suggest early cortical stages of visual processing are compromised for adult ADHD	16 (good)
Liechti, 2013, Switzerland (33)	Resting EEG during separate 3-min eyes open and eyes closed conditions, followed by cognitive ERP tests including cued continuous performance tests. 10–20 system 48-channel EEG. Within and Between group comparisons	Adult ADHD group age range 32–55.	*n* = 54, ADHD (Children (8–16 years)) = 32, (Adults) = 22, controls = (Children) = 30 (Adults) = 21	No consistent theta or theta/beta increases were found in ADHD. Even multivariate analyses indicated only marginal EEG power increases in children with ADHD. Instead, consistent developmental theta decreases were observed, indicating that maturational lags of fewer than 3 years would have been detected in children.	18 (excellent)
Loo, 2013, US (34)	Resting EEG, 5-min eyes closed and eyes open conditions. ADHD versus control and between age group analysis. 40 Ag/AgCl surface electrodes in an extended international 10/20 location system.	Excluded if current comorbid psychiatric disorders, IQ<80. Adult age range 32–64 years.	*n* = 871 ADHD = 309, control = 562 (collectively)	Theta-Beta ratio did not differ significantly by ADHD status for youth but was significantly lower in adults with ADHD compared with controls. ADHD subtype and psychiatric comorbidities such as disruptive behavior disorders and depression have opposing and significant mediating effects on the TBR.	17 (good)
Markovska-Simoska, 2017, Macedonia (35)	EEG was recorded during an eyes-open condition. Spectral analysis of absolute and relative power. Electrode cap with 19 electrodes using the 10–20 system	ADHD adults mean age 35. 8 years. Comorbid diagnoses were excluded from the analysis. None of the subjects had any serious medical or neurological problems (including seizures) or recent (<6 months) head trauma. No one from the examined individuals was taking psychostimulants	*n* = 120 ADHD = 30 (male) children, 30 (male) adults, control = 60 (matched by age)	ADHD children showed increased absolute power of slow waves (theta and delta), whereas adults exhibited no differences compared with normal subjects. Relative power spectra showed no differences between the ADHD and control groups. Only ADHD children showed greater TBR compared to the normal group. Classification analysis showed that ADHD children could be differentiated from the control group by the absolute theta values and theta/beta ratio at Cz, but this was not the case with ADHD adults	16 (good)
Marquardt, 2018, Norway (36)	ERP study. Participants performed modified version of the Eriksen flanker task. EEG recorded using a 64-channel equidistant electrode cap with Ag/AgCl electrodes. The recording reference was placed at Cz, with a ground placed at approximately AFz	ADHD group mean age 35.4 years. All ADHD participants had a formal diagnosis according to national standards. Groups did not differ regarding IQ, sex, age, or handedness. Comorbidities were not excluded in either the ADHD or control group; anxiety /depression (*n* = 18), autism spectrum disorders (*n* = 3), bipolar disorder (*n* = 2), alcohol-related problems (*n* = 2), drug-related problems (*n* = 2), treatment for other mental health problems (*n* = 8), and eating disorders (*n* = 4). Participants taking stimulants were asked to refrain from medication for 48 h prior to investigation	*n* = 63 ADHD = 31, control = 32	Behavioral measures did not differ between groups. However results from P3, ERN, and Pe suggest persistent alterations of attentional and error-monitoring processes for ADHD adults. Further, ERP component amplitudes and behavioral ACC correlated with ASRS scores, thus further suggesting a correspondence between electrophysiological measures and ADHD symptoms	19 (excellent)
Missonnier, 2013, Switzerland (37)	Continuous EEG recorded using 20 surface electrodes. Locations, according to the 10–20 international system. Each group performed two tasks. Between group analysis	Excluded neurologic conditions. ADHD mean age 34.3 years, age range 26–40	*n* = 30 ADHD = 15, control = 15	ADHD patients displayed lower alpha event-related de-synchronization compared to controls	19 (excellent)
Poil, 2014, Switzerland (38)	2.5-min resting state recording with eyes closed position. 60 electrodes on scalp. Using 10–20 system, plus a number of 10-10 positions	The subject groups were matched for handedness, gender, and estimated IQ. The control subjects did not report any current or previous neurological or psychiatric diagnoses	*n* = 116 ADHD = 48 (22 adults), control = 68 (27 adults)	Adult ADHD demonstrated slowing of alpha frequency, combined with a higher power in alpha and beta. Main findings suggest effects of ADHD on EEG are age dependant	17 (good)
Ponomarev, 2014, Russia (39)	Resting EEG, CSD and gIC analysis (19 channels, linked ears reference, eyes open/closed). Between group comparison	Excluded complex perinatal period, head injury with cerebral symptoms, history of neurological or psychiatric diseases, convulsions, nonnormal mental and physical development, below average or better grades in school, current medication or drugs. ADHD group mean age 36.4 with a range of 20–50	*n* = 472 ADHD = 96, control = 376	Pattern of differences in gIC and CSD spectral power between conditions was approximately similar, whereas it was more widely spatially distributed for EEG. Size effect of differences in gIC and CSD spectral power between groups of subjects was considerably greater than in the case of EEG. Significant reduction of gIC and CSD spectral power depending on conditions was found in ADHD. Reducing power in a wide frequency range in the fronto-central areas is a common phenomenon regardless of whether the eyes were open or closed. Spectral power of local EEG activity isolated by gICA/CSD may provide a discriminating biomarker	18 (excellent)
Strauß, 2018, Germany (18)	Cross-sectional study. Assessed brain arousal regulation using the VIGALL 2.1 to analyze 15-min resting EEG data. Cap mounted, using 31 electrodes and the 10–20 system. Eyes closed position	Mean age 30.5. Exclusion criteria: organic mental disorders, current (moderate/severe) depressive episode or subthreshold depression, acute suicidality, current manic episode, bipolar disorder, schizophrenia or psychosis; current somatic disease, use of psychotropic medication, EEGs containing more than 15% artifacts or 95% low voltage segments	*n* = 68 ADHD = 33, control = 35	Adult ADHD patients showed significantly lower arousal levels and significantly less stable brain arousal regulation than controls	18 (excellent)
Tombor, 2018, Hungary (40)	Spontaneous EEG was recorded with eyes open. 4-min resting state. Focussed on low gamma frequency band, ranging between 30 and 48 Hz. Between subjects design	Subjects were individually matched by age, gender and level of education. The inclusion criterion for the patient group was the diagnosis of ADHD. Exclusion criteria for all participants included a history of severe neurological or somatic disorder or severe head trauma	*n* = 101 ADHD = 42 (methylphenidate naïve), 17 (methylphenidate treatment), control = 42	ADHD showed lower task-free gamma band activity compared to controls. Manifest in the right hemispheric and midlne centroparietal areas. Lower right central and centroparietal activity (30.25–39Hz^)^, was associated with ADHD severity. Inverse association found between right centroparietal (39.25-48Hz) activity and ADHD severity. Findings irrespective of medication	18 (excellent)
Woltering, 2012, Canada (41)	Resting-state EEG with eyes-open and eyes-closed conditions, were compared for college students with ADHD and nonclinical control. 129 channel EEG net. Between group analysis	Excluded those with uncorrected sensory impairment, major neurological dysfunction and psychosis, and current use of sedating or mood altering medication other than stimulants prescribed for ADHD. Mean age of 25.8 years	*n* =35 ADHD = 18, control = 17	ADHD group showed decreased power for fast frequencies, especially alpha. Also increased power in the slow frequency bands, however, these effects were strongest using relative power computations. Furthermore, the TBR measure was reliably higher for the ADHD group. All effects were more pronounced for the eyes-closed compared to the eyes-open condition. Measures of intra-individual variability suggested that brains of the ADHD group were less variable than those of controls	15 (good)

*Employed data set from Hale et al. (26).

## Results

Of the 21 studies included, the median number of participants was 34 (excluding control groups), with a range of 295. The largest study included 309 participants, the smallest 14. The mean age of participants (adult subgroups only) was 33.7 (*SD ±* 7.05) years. Study design varied significantly. From the twenty-one studies included, only one did not employ a control group (24). It was however included because it met inclusion criteria. **Table 1** provides additional information about the nature of each study. In terms of quality, 42.9% of studies included in the review were rated excellent, 52.3% good, 4.8% fair, and 0% were rated poor. The median quality score was 17, with a range of 13 to 19. Of the studies included in this review, evidence for elevated levels of both absolute and relative theta power and alpha activity, along with a general reduction in neural activity for tasks requiring attention in adult ADHD populations was established, however there was variance in study design and inconsistency of findings. To a lesser degree, no differences are evident for delta or beta activity, and no consistent evidence of atypical TBR in adult ADHD. One study reported reduced resting state gamma activity. Results also support reduced CNV amplitude as a possible biomarker for adult ADHD (see **Table 1**).

## Discussion

An objective, reliable measure to aid diagnosis would be extremely valuable, in any clinical setting. Such a tool would allow for accuracy in diagnosis, be economical and ensure consistency. Such would be the scope in the use of EEG to aid adult ADHD diagnosis should the evidence support that there are patterns specific to adult ADHD. However, the summative narratives of the studies included in this review suggest that observable EEG activity that is unique to adult ADHD is consistently variable.

For instance, consistent reporting of elevated theta (4–8 Hz) levels for adult ADHD would be usually associated with focused attention and difficulty of task. Indeed, the studies show evidence for elevated absolute and relative power in adult ADHD populations; however, these observations are seemingly dependent on the developmental phase hence not consistent. For example, there is evidence for elevated theta waves “at rest” comparative to control subjects, as well as observed elevated theta in Event Related Brain Potentials (ERP) analysis. Further to this, quantitative EEG allowed differentiation between not only adults with ADHD and controls, but also those with ADHD symptoms who did not meet the criteria to be given a diagnosis of ADHD, demonstrating the potential sensitivity that would be required in order to use EEG for diagnostic purposes (23). This suggests adequate support for the notion that theta activity is typically elevated in this population. However, at the same time, Liechti et al. (33) failed to find consistent elevated theta waves (however the sample of 22 participants could be considered relatively small), and Markovska-Simoska & Pop-Jordanova (35) only observed elevated theta in children, not adults. However taking lead from the longitudinal study by Feinberg, De Bie, Davis, & Campbell (42), they suggest a decline in EEG power is likely a reflective of brain reorganization which is driven by synaptic pruning. They discovered that slower waves decreased by up to 60% in ages 11 to 17 years, which could explain why patterns of inconsistency between ADHD in childhood comparative to adulthood are observed. Nevertheless, while the majority of studies tend to report elevated theta this is not a consistent trend and therefore cannot fully be endorsed as a specific biomarker, as yet.

Collectively, it is difficult to synthesize the findings in relation to alpha waves, as again, inconsistency is apparent in the findings of the studies included in this review. In summary, there is evidence of increased absolute power and decreased relative power to alpha waves alongside slower frequency in adulthood ADHD. More specifically, in terms of “at rest” studies, there is evidence of decreased alpha activity, and increased alpha activity. For instance, Poil et al. (38) found that in adults with ADHD there was slowing of alpha frequency along with increased power in alpha-1 (8–10 Hz) when eyes were closed for resting, but this resting state EEG activity was altered in ADHD in relation to age, suggesting that ADHD subjects clearly show a different maturation profile comparative to neurotypical populations; this has already been noted elsewhere (43). On the other hand, Woltering, Jung, Liu, & Tannock (41) report a decreased alpha power at rest. Reconciliation of these findings is difficult to attain here, with the eyes open/closed positioning not an explanatory factor in these findings.

As for beta waves (12–25 Hz), which are typically higher when a person is active, busy, anxious or concentrating for example, it is apparent that beta activity patterns are not unique in adult ADHD profiles and cannot be useful in differentiating ADHD in adulthood from normative populations based on evidence included in this review (23, 31). Yet, notably, there is evidence of abnormal rightward beta asymmetry in inferior parietal regions, which is potentially an important feature in adult ADHD which may occur due to impaired capacity for top down task directed control over sensory encoding functions associated with attention (26, 27). This insight provides intriguing information in relation to how EEG could be employed in ADHD populations, however at this point in time, beta activity is unable to offer reliable information in terms of differentiation of ADHD populations and more research is required to offer clarification.

Furthermore, a similar picture emerges when considering the information available regarding delta waves; there is no clear narrative regarding a potentially unique pattern of delta activity for adult ADHD populations when considering the information derived from this review, which suggests that, as yet, delta activity is not insightful for this population.

In terms of gamma activity, Tombor et al. (40) reports the first study to investigate resting state gamma band activity, finding this wave reduced (ranging from 30 to 39Hz) in adult ADHD, predominantly in the right hemispheric and midline centroparietal areas, comparative to controls. Curiously, gamma bands between 39 and 48 Hz were inversely correlated with ADHD severity, suggesting that reductions in gamma are associated with ADHD, perhaps reflecting dysregulation of neural networks associated with attention. This observation is in line with childhood studies which have found reductions in gamma activity compared to controls (44, 45). Tombor et al. (40) suggest their findings propose the dysregulation of neural networks relating to attention capacity, yet what limits the conclusions of this study is that it only employed an eyes open condition; therefore we cannot compare the two conditions. Also, the effect of medication on the sample is unknown but it does offer interesting information as to how gamma band is potentially insightful in relation to ADHD and EEG, albeit requires more research.

In general, the observed cortical under arousal reflected in the decreased contingent negative variation (CNV) in the EEG waves supports the notion of a reduced CNV amplitude as a possible biomarker for ADHD (46). This pattern of results with regards to EEG waves may be explained by the hypoarousal model of ADHD as a consequence of low tonic dopamine levels and the subsequent up-regulation of autoreceptors (47).

Considering reports from ERP studies, a collective evaluation shows slowing or reduced EEG activity in adults with ADHD, comparative to controls. EPRs are a small section of the continuous EEG recording, which have been induced by a response to cognitive processing, such as viewing of stimuli during a test battery. ERP studies are particularly useful in exploring the evoked neurological response to specific cognition. The recording will demonstrate the electrical activity for a particular task at a particular moment in time. Of the studies included in this review, Leroy et al. (32) in a visual attention study, found a decrease in neural networks in adult ADHD, suggesting compromised early cortical stages of visual processing. Herrmann et al. (29) reported the first study looking at neural correlates of error processing, finding post error slowing in adults (mean age of 24.2 years) but not observed in an older sub group of adult ADHD (mean age of 40.9 years). This suggests ADHD can be characterized by abnormal ERP of the error positivity (Pe), however this abnormality seemingly vanishes with age. Gonen-Yaacovi et al. (25) found neural response variability was lager in ADHD participants compared to controls for visual and auditory stimuli, but results also show neural variability throughout the task, suggesting larger activity is not related to specific cognitive processes and rather, continuous. However, it must be acknowledged that this study was limited in that it had a small sample size. Further, Hasler et al. (28) observed reduced activation of the functional networks devoted to bottom-up and top-down attention, which suggests adults with ADHD have reduced cortical resources for tasks related to these processes. Hale et al. (26, 27) found ADHD to have atypical right lateralization of brain function during a continuous performance test (a test that ADHD populations consistently show impairment on) in inferior parietal brain regions, an area linked to ADHD by other studies (48). This is relevant for ADHD treatment as normalizing lateralized contributions have worked previously for dyslexia patients (49). In accordance with what would be expected, it is evident that increased variability and a slowing of cortical activity are found in adult ADHD. However, there is also evidence to suggest that these observations are potentially confounded, especially by maturation.

### Theta/Beta Ratio

Based on our discussion relating to the changes to theta and beta waves in ADHD, we would expect to find some results relating to TBR namely elevation as there is increase in theta waves and rightward beta asymmetry in inferior parietal regions. Indeed this knowledge has been used to develop a commercial product called Neuropsychiatric Electroencephalograph-Based ADHD Assessment Aid (NEBA) system, which received FDA approval (Food and Drug Administration, 2013) (50) for childhood populations. The TBR is considered ‘robust’ especially when considering the quantity of studies supporting this model, and the finding that medication specific to ADHD stabilizes TBR in ADHD patients (51). It is not however clear how this would translate to adult populations. In this review, we found studies reporting no consistent evidence of atypical TBR in adult ADHD, despite the evidence in childhood samples (17). However, it must be considered that the irregularity in findings might be explained in the context of sampling issues. Specifically, Loo et al. (34) found that the TBR was reversed in ADHD, in that it was significantly lower comparative to controls, yet they state that included within the non-ADHD adult sample was parents of children included in the ADHD group. It is therefore possible that the non-ADHD adult group possessed a strong loading of gene variants and traits associated with ADHD. Moreover, Loo et al. (34) acknowledge that a majority of the non-ADHD adult group had a range of other psychiatric diagnosis which might account for the inverse ratio. It is therefore possible that the non-ADHD adult group possessed a strong loading of gene variants and traits associated with ADHD. Moreover, the majority of the non-ADHD adult group had a range of other psychiatric diagnosis which might account for the inverse ratio. In addition, Liechti et al. (33) found that TBR increase was not present in ADHD, even when multivariate analysis was applied.

### EEG and Brain Maturation

A potential important explanation for the discrepancy in EEG activity in adult ADHD comes from the issue of the differences in neurological activity according to maturation. It seems there is evidence to suggest that the TBR is more reliable in childhood (50), albeit with some issues as use of fixed EEG bands may be a significant limitation, particularly in youth (52). Evidence points to a combination of reduction of TBR in ADHD as patients grow older, alongside the increase in TBR as neurotypical populations increase in age (53). This is likely related to the reduction in hyperactivity in adulthood ADHD, linked to frontocentral normalization of beta activity, and maintained increase in theta activity associated with impulsivity (54). In support of this line of thinking, Koehler et al. (31) found absolute power density in alpha and theta bands (posterior region only) to be significantly increased in adults with ADHD compared to matched controls when measuring resting state in an eyes closed position. No differences were found for delta and beta activity, with authors suggesting they could not be used to discriminate ADHD from neurotypical presentation. Increased alpha activity was found in frontal, central, and posterior regions of the brain. In addition, theta power and age shared a negative correlation, demonstrating again that age is a significant confounding factor in EEG abnormality in adult ADHD. They conclude that ADHD cortical activity is different in adults with ADHD compared to younger populations, and that EEG abnormalities change with maturation and suggest longitudinal research should be carried out to determine why this is observed.

Neurotypical populations consistently present with a decrease in theta activity and an increase in alpha activity as development progresses (55), with EEG frequency increasing in a linear relationship with maturation (56). For ADHD where it has been proposed that the brain maturation process is affected (4), this would introduce an important confounder. This is along the lines of what is reported by Poil et al. (38) who found that the effect of ADHD on EEG is greatly dependent on age.

The notion of age is a seemingly complex one, and a confounding factor that requires clarity if EEG is to be useful in a clinical settings. van Dongen-Boomsma et al. (57) suggest that discrepancies between childhood and adult ADHD EEG findings may be because of a multitude of factors, one being study design. They suggest that the majority of childhood studies look at eyes closed data, while the majority of adult studies look at eyes open conditions (here, we find a mixture of both) and perhaps this could explain some of the discrepancy. A previous meta-analysis looking at ADHD in childhood, adolescence and adulthood found consistent theta power increase and beta power decrease, also that the TBR in ADHD is commonly increased, and can predict ADHD with 94% sensitivity (58). We think this is a very ambitious conclusion particularly in relation to the findings here which cannot support such consistency in adults. What is interesting about this meta-analysis is that while they conclude consistency in EEG activity in ADHD, they also suggest that age related changes significantly affect the TBR in ADHD.

### EEG Analysis

When Ponomarev, Mueller, Candrian, Grin-Yatsenko, & Kropotov (54) investigated the performance of spectral analysis of resting EEG by calculating current source density (CSD) and group independent component analysis (gICA) in 96 adults with a diagnosis of ADHD, they found that these measures were more sensitive in distinguishing ADHD from control populations in comparison to raw EEG data in the front-central areas. Furthermore, Biederman et al. (22) report an EPR study which employed brain network activation (BNA) analysis to achieve qualitative data on cortical connectivity to EEG data that was collected during a Go/No-Go task in adults with and without ADHD. They suggest BNA analysis produces an algorithm which is able to discriminate between ADHD and normative groups with a high level of accuracy. Moreover, the BNA ADHD profile is able to be refined further to build individual neural network profiles that are typical for subgroups that could be characterized by age and gender; a prospect that would be highly valuable. This approach is in line with Ahmadlou & Adeli (59) who previously suggested that ADHD diagnosis using EEG should use wavelets, a signal processing technique and neural networks, a pattern recognition technique as the signal is often chaotic and complex. They suggested that using nonlinear, multiparadigm methods would yield the most accurate results to aid diagnosis. Using a wavelet-synchronization method they reported a 95.6% accuracy of diagnosis in a sample of 47 ADHD patients. Therefore, it seems imperative to continue this trend of theory driven research that has the potential to establish a robust measure of ADHD using EEG in adult populations.

### Future Research

Clearly, EEG as a diagnostic tool for adults with ADHD has discrepancies that require clarification. Leading themes here have surrounded the complexity of the maturation process and how this is a confounder in establishing a clear narrative of how EEG can be useful in adult ADHD. Further, the describing of raw EEG data without a theory driven study and standardized protocol, is problematic. It is possible that this approach is inhibiting the development of useful information regarding this potentially valuable method to aid diagnosis. A call for longitudinal research is required, mainly to unpick how cortical activity changes in this atypical population and how EEG can help us determine this. Also, mediating factors such as comorbidity and ADHD heterogeneity can significantly influence the inconsistencies found in these studies. This highlights that method is extremely important in EEG studies, in that age, subtype of ADHD and comorbidity need to be accounted for, otherwise, we should expect to see differing and contradictory results. Again, this observation links to our conclusions surrounding age and its’ effect on the value of EEG measures. Replication is needed to gain consistency across findings for particular waves, within this, research should be theory driven, rather than describing EEG data patterns. Finally, there is evidence to suggest that EEG abnormalities in ADHD are influenced by biological sex in childhood (45), a concept not approached in the studies available to this review, but one that requires further investigation to determine if sex differences could account for some of these inconsistencies.

## Conclusions

There is potential the EEG can be used as a clinical diagnostic tool in Adult ADHD. However, with the current level of knowledge, it cannot be recommended. Although most studies point to elevated levels of both absolute and relative *theta power* and *alpha activity* as having potential to differentiate between adult ADHD and normative populations along with a general reduction in neural activity for tasks requiring attention, these findings are not consistent among studies to recommend it use.

In addition, the studies included in this review used differing paradigms and different samples so the exact nature of the EEG profile in adult ADHD remains unclear. However, one observation that is steadfast is the discrepancy in findings when comparing childhood and adulthood EEG activity which indicates developmental changes are atypical in ADHD.

It is also clear that the once considered robust measure, TBR, is highly inconsistent in adult ADHD and a new wave of research that considers and adds clarity to these discrepancies is required in order for this potentially valuable measure of ADHD in adulthood to be introduced.

## Data Availability Statement

The original contributions presented in the study are included in the article/supplementary material; further inquiries can be directed to the corresponding author.

## Author Contributions

MA, TF, and SJ contributed equally. All authors contributed to the article and approved the submitted version.

## Conflict of Interest

The authors declare that the research was conducted in the absence of any commercial or financial relationships that could be construed as a potential conflict of interest.

## Abbreviations

ACC, reduced accuracy; ADHD, attention deficit hyperactivity disorder; ASD, autism spectrum disorder; ASRS, adult self-report scale; BNA, brain network activation; CPT, continuous performance task; CSD, current source density; CVN, contingent negative variation; DSM-IV, Diagnostic and Statistical Manual of Mental Disorders, 4th. Edition; EEG, electroencephalography; ERN, error-related negativity; ERP, event related potential; FDA, Food and Drug Administration; GFS, global field synchronization; gIC/gICA, group independent component analysis; Go/NoGo, response inhibition task; NEBA, neuropsychiatric electroencephalograph based ADHD assessment aid; Pe, error positivity; qEEG, quantitative electroencephalography; TBR, theta/beta ratio; VIGALL, Vigilance Algorithm Leipzig; VLF, very low frequency.
